# DOES DEPRESSION EXPLAIN COGNITIVE CHANGES IN PSYCHOTHERAPY? A MEDIATION ANALYSIS

**DOI:** 10.1093/geroni/igae098.2671

**Published:** 2024-12-31

**Authors:** Emily Post, Teresa Walker, Matthew Schurr, Brenna Renn

**Affiliations:** University of Nevada Las Vegas, Las Vegas, Nevada, United States; University of Nevada Las Vegas, Las Vegas, Nevada, United States; University of Nevada Las Vegas, Las Vegas, Nevada, United States; University of Nevada Las Vegas, Las Vegas, Nevada, United States

## Abstract

Depression commonly affects older adults and is associated with detrimental outcomes, including cognitive impairment such as executive dysfunction. Psychotherapy effectively improves depression outcomes and may improve some aspects of cognition, but this relationship is not widely understood. This study examined the potential mediating role that depression severity might have between psychotherapy treatment and a change in cognitive test scores. Data for this secondary analysis were drawn from older adult participants (N=249, mean age = 70.2 [7.4] years) with major depressive disorder who participated in a RCT comparing two 9-week structured psychotherapies for depression. Our prior work found improvement in depression (noninferiority between treatment groups) and some improvement on measures of cognition during the RCT. Measures included pre- and post-treatment cognitive tests: Iowa Gambling Money Task, Digit Span Backwards, and Stroop test. The Hamilton Depression Rating Scale (HAM-D) assessed change in depression symptoms. Hayes PROCESS macro for SPSS analyzed separate mediation models testing depression as a mediator of treatment and each cognitive outcome. Change in depression did not mediate relationships between treatment and change in cognitive outcomes across the nine weeks of psychotherapy. While our investigation did not establish depression as a mediator of change in cognitive test performance in patients receiving psychotherapy, these findings may indicate that improvement in depression is somewhat independent of any observed changes in cognitive test performance. Future research involving a more cognitively impaired sample could strengthen these findings.

